# Synthesis, docking studies, biological activity of carbon monoxide release molecules based on coumarin derivatives

**DOI:** 10.3389/fchem.2022.996079

**Published:** 2022-09-29

**Authors:** Huapeng Liu, Yujie Chen, Fujun Cui, Yuan Liao, Xicun Wang

**Affiliations:** ^1^ College of Medical Engineering and the Key Laboratory for Medical Functional Nanomaterials, Jining Medical University, Jining, China; ^2^ College of Chemistry and Chemical Engineering, Northwest Normal University, Lanzhou, China; ^3^ Gansu Police Vocational College, Lanzhou, China

**Keywords:** co-releasing molecules, coumarin derivatives, docking studies, drug-like properties, antitumor activity

## Abstract

In the present work, we synthesized seven complexes. All complexes were identified by ESI-HRMS, ^1^H-NMR, ^19^F-NMR and ^13^C-NMR spectroscopies. The synthesized complexes were tested for their anticancer activities *in vitro* against three different human cell lines, including breast MDAMB231, cervical Hela, liver HepG2. IC_50_ values of complexes 1-7 were 34.98–667.35 µM. Complex 5 revealed higher sensitivity towards MDAMB231 cells with IC_50_ values 34.98 μM in comparison to 5-FU as positive control. Moreover, complex 5 caused a decrease of mitochondrial membrane potential and effectively induced ROS production against MDAMB231 cells. Western blot analysis showed that complex 5 could up-regulate the expression of Bax protein and down-regulate the expression of Bcl-2, activate Caspase-3, slightly down-regulate the expression of HO-1. The docking studies showed that complex 5 could be interacted with Bcl-2 protein through hydrophobic interactions, hydrogen bonds and salt bridges to enhance the binding affinity. All the analyzed coumarins obeyed the Lipinski’s rule of five for orally administered drugs. Based on the aforementioned results, it suggests that the complex induced apoptosis cell *via* mitochondria pathways. Collectively, complex 5 could be considered as a promising hit for new anti-breast cancer agents. Carbonyl cobalt CORMs, as potential anticancer therapeutic agents, provided a new idea for the development of metal anticancer drugs.

## Introduction

Cancer is one of fatal diseases that caused millions of deaths annually (Yoon et al., 2022). Prostate, lung and bronchus, and colorectal cancers (CRC) account for almost one-half of all new cases in men. For women, breast cancer, lung cancer, and CRC are responsible for 51% of all cancer deaths, with breast cancer alone accounting for almost one-third (Siegel et al., 2022). Reasons for higher cancer risk in men are not fully perceptible but probably react more exposure to cancer-causing environmental, occupational and biological risk factors. Sex differences in endogenous hormones and immune function may also play a significant role in different cancers (De Nardi et al., 2022). In the current cancer treatment, chemotherapy is still the dominant treatment strategy (Network, 2012). The chemotherapeutic anticancer drugs are highly toxic, such as the nephrotoxicity of cisplatin (Huang et al., 2022), liver toxicity of carmustine (Ricciardi et al., 2022). The development of low toxic and effective anticancer drugs could be the main goal of novel drug research and development (Elshemy and Zaki, 2017).

Coumarin compounds are containing 1, 2 benzopyranone structure, most of which were extracted and isolated from plants (Thakur et al., 2015). Coumarins have unique physical and chemical properties, such as π-π conjugation, rich in electrons and charge transfer properties. Which could bind to many targeted proteins (Venkata Sairam et al., 2016; Srikrishna et al., 2018). Coumarins derivatives could exhibit biological activities such as the anti-inflammatory (Bansal et al., 2013), antibacterial (Qin et al., 2020), antiviral (Zhao et al., 2019), anti-clotting (Kidane et al., 2004) and anticancer (Chilin et al., 2008; Emami and Dadashpour, 2015; Garro et al., 2016). At present, structural modifications of Coumarin may provide the potent cancer cell growth inhibitory activity at 3, 4, and 7-position of benzopyranone. Complexes of zirconium (IV), zinc (II), and cadmium (II) based on a substituent at 3-position of coumarin demonstrated potent antitumor activity *in vitro* (Kostova et al., 2008). The introduction of substituents at position 4 could change the biological activity of the drug. Interestingly, the substitution of position C-4 on pyran ring possessed significant anticancer activity (Karaivanova et al., 1994; Kumar et al., 2005). The compounds modified with 7-hydroxycoumarin also showed great inhibitory activity against tumor cells (Konc et al., 2011). Modifying coumarin derivatives to construct new chemical targeting entities with strong pharmacological activity had become an important research direction in the field of drug development. A series of bi-functional platinum (IV) complexes with 7-hydroxycoumarin ligands were designed and synthesized. Those complexes exhibited higher cytotoxicity compared to cisplatin due to its targeting property and to be effectively introduced into the cell through coumarin ligand. The combination of metal and coumarin derivatives increases cell intake and improves biological activity (Balcioglu et al., 2020). Iridium(I) complexes bearing hemilabile coumarin-functionalised N-heterocyclic carbene ligands could enhance biological activity (Karatas et al., 2021). The modified complexes at 6,7-position and 3,4-position on benzopyranone showed a remarkable cytotoxic activity (Bertrand et al., 2014; Brinda et al., 2019; Balewski et al., 2021). Additionally, it suggests that concentrating on relatively safe metals such as cobalt, gold, silver, copper, and iron would be more beneficial in the field of low side effects.

Metal carbonyl complexes, as carbon monoxide releasing molecules, were typically used to deliver a well-controlled dosage of carbon monoxide to cells and tissues. In the past decade, people have synthesized several different types of carbon monoxide releasing molecules (CORMs) and evaluated their potential applications in clinical medicine (Motterlini and Otterbein, 2010; Romão et al., 2012). In particular, carbonyl cobalt carbon monoxide release molecules have shown potential inhibition against cancer cell lines (Riveiro et al., 2008). The combination of carbonyl cobalt carbon monoxide release molecules with pharmacological ligands could significantly improve the anticancer activity (Gong et al., 2015; Li et al., 2018; Liu et al., 2021). It was well known that the uncoupling of mitochondrial function could lead to the increase of intracellular superoxide (Li et al., 2021). Herein, a series of complexes were synthesized by combining coumarin derivatives with carbonyl cobalt carbon monoxide releasing molecules for antitumor applications. In addition, mechanism of the complex, molecular docking studies, ADME evaluation studies for all synthesized complexes were studied.

## Marerials and methods

### Reagents and instruments

All chemical reagents were commercially available and used as received unless otherwise stated. All reactions were carried under nitrogen atmosphere. Solvents for reactions were distilled from the proper drying agents. Column chromatography was carried out using 200–300 mesh silica gel and Neutral alumina. Tumor cell lines were from Center for Shanghai Life Science Institute of Chinese Academy of Sciences (China). IR spectra were recorded on a DIGILAB FTS-3000 spectrophotometer, NMR was recorded on Varian Mercury plus-400 MHz and Varian Mercury plus-600 MHz. HRMS data were carried out on a Thermo Scientific Q exactive.

### Cell culture

The cells were cultured with 100% humidity in RPMI1640 medium and supplemented with 10% fetal bovine serum. Cell lines were incubated at 37°C under 5% CO_2_ atmosphere.

### MTT assay for *in vitro* cytotoxicity

Growth inhibitory effect toward cancer cells line was determined by means of MTT colorimetric assay (Gerlier. and Thomasset, 1986). The tumor cells were seeded in 96-well plates at 5 × 10^3^ cells per well. The tumor HepG2, Hela, MDAMB231 cells were treated with 10 μM–150 μM of the complex using 5-Fu as the positive control for 24 h, respectively. After incubation, culture medium was removed and 20 μL of MTT reagent was added to each well. The formazan product was dissolved by addition of 100 μL DMSO per well. Optical density (OD) values of the purple solution that represented cell viability were measured at 570 nm. The cell viability was evaluated by measuring at a wavelength of 490 nm (Spectra Max, United States) with a microplate reader.

### Detection of ROS

MDAMB231 cells were seeded into 6-well plates and incubated with complex 5 (0, 40 μM, 80 μM) at 37°C for 24 h. The cells were collected by centrifugation, washed and incubated at 37°C with incomplete growth medium containing the DCFH-DA for 30 min in the dark. Subsequently, the mediums were removed and washed with DMEM medium three times. Then the cells were suspended in incomplete growth medium. The cells were photographed by OLYMPUS IX73 microscope with excitation wavelength at 488 nm.

### Assessing mitochondrial membrane potential

MDAMB231 cells were seeded in 6-well plates at a density of 1 × 10^4^ cells/well for 24 h. The cells treated with complex 5 for 24 h at the specified concentration (0, 40 μM, 80 μM). Then, the culture medium was discarded, and cells were stained with a JC-1 dye at 37°C for 15 min. Finally, the samples were collected and imaged by OLYMPUS IX73 microscope.

### Protein extraction and western blot analysis

MDAMB231 cells were seeded into culture flask. After incubation for 24 h, the cells were treated with complex 5 (10 μM–120 μM) for 24 h. Then the cell lysate was obtained and the protein concentration was measured. The same amount of protein (20 μg) was separated from 10% SDS-PAGE gel and transferred to polyvinylidene fluoride (PVDF) membrane (MilliporeCorporation, United States). The imprinting was sealed in a closed buffer at room temperature for 2 h, and then incubated with the diluted solution (1:500–1:1000) of Caspase-3 (abcam, 96625), Bax (genetex, 109683), Bcl-2 (genetex, 100629), HO-1 (abcam, 68477). The antibody (US) blocked the buffer overnight at 4°C. Then the membranes were incubated with the appropriate secondary antibody (1:5000–1:10000 dilution), and GAPDH (immunoway, B1501) was used as a loading control. The protein bands were visualized by X-ray dark box (11–14 of Shantou Yongtai Medical Instrument Co., Ltd.).

### Molecular modeling

In this project, we mainly use autodockvina program for molecular docking (Trott and Olson, 2010). The structure file of the target protein Bcl-2 is derived from the Protein Date Bank (PDB ID:2XA0, resolution = 2.70) and the structure file of the complex 5 is drawn by Chem3D software. The structure files of target proteins and target small molecules was carried out with AutoDock Tools software, such as adding H atom, adding Gasteiger-Hücker empirical charge, merging non-polar hydrogen,setting rotatable bond and so on. Among them, the σ bonds between heavy atoms in the target small molecular structure are set as rotatable bonds, while the rest of the target protein Bcl-2 is regarded as a rigid structure. During docking, the AutoDock Vina program carried out conformational searchand energy optimization in the square box (38 × 38 × 38) set at the active site of the target protein Bcl-2. The calculation was terminated after the best binding conformation was obtained, and the other parameters used to run the AutoDock Vina program were the default values. Finally, we will use Protein-Ligand Interaction Profiler online server (https://projects.biotec.tu-dresden.de/plip-web/plip/index) to analyze the intermolecular interaction of the best binding conformation, extract the key interaction parameters such as bond length and angle, and draw a three-dimensional interaction diagram by Pymol software.

## Synthesis of the ligands

### A-G

3-carboxylic acid*-*2H-Chromen-2-one (A): The raw material A was synthesized according to the literature method (Taheri et al., 2019). Salicylaldehyde (20 mmol) and meldrum’s acid (22 mmol) were dissolved in 40 ml water and refluxed the mixture for 15 h. The solution was then cooled, filtered and dried to obtain a white solid with a yield of 92%. mp: 188–189°C.

7-Diethylamino-3-carboxylic acid-2H-Chromen-2-one (B): The raw material B was synthesized according to the literature method (Draoui et al., 2013). 4-(diethylamino)-2-hydroxybenzaldehyde (5.18 mM), meldrum’s acid (5.18 mM), piperidine (0.52 mM) and two drops of acetic acid was dissolved in ethanol (8 ml) and stirred for 30 min at room temperature and refluxed for 3 h. The reaction mixture was poured onto 15 ml of ice water. The target product was collected and washed with ethanol to give an orange crystalline solid (Yield: 90%). mp: 150–153°C.

7-hydoxy-2H-Chromen-2-one (C) and 7-hydoxy-3-Acetyl--2H-Chromen-2-one (D) have been prepared *via* Knoevenagel condensation reaction. Compounds C and D were synthesized following the same procedure as for the literature method (Reddy et al., 2004). C: 10 mmol of 2,4-dihydroxybenzaldehyde and 10 mmol of Malonic acid are dissolved in 250 ml of ethanol. After adding of appropriate piperidine, the mixture was stirred at reflux for 2 h. Then, the solvent was removed by rotary evaporation to obtain the crude product, which was further purified by column chromatography using ethyl acetate as eluent;7-hydoxy-3-Acetyl--2H-Chromen-2-one (D) were also prepared by reacting 2,4-dihydroxybenzaldehyde with ethyl acetoacetate in the presence of piperidine and ethanol.

7-hydoxy-4-methyl-2H-Chromen-2-one (E): The raw material E was synthesized according to the literature method (Najjar and Bigdeli, 2018). In a 100 ml round bottom flask, cool 25 ml of concentrated sulfuric acid to 10°C in an ice bath. The acid was added drop by drop to the solution of resorcinol (0.55 g, 5 mmol) and ethyl acetoacetate (0.71 g, 5 mmol) in 2 h. Then, the reaction mixture was stirred for 16 h at room temperature. Finally, the reaction mixture was poured onto the crushed ice. The separated yellowish solids were filtered out. The solids were dispersed in 5% sodium hydroxide solution, and then the separated solids were filtered and washed with ice water. Yield: 90%. mp:174–177°C. The synthesis methods of F and G were similar to that of E.

7-hydoxy-4-Difluoromethyl-2H-Chromen-2-one (F): Yield: 20%. mp: 72–74°C.

7-hydoxy-4-trifluoromethyl-2h-Chromen-2-one (G): Yield: 83%. mp: 191–193°C

### A1-G1

2-Oxo-N-2-propyn-1-yl-3-carboxamide-2h-chromen-2-one (A1): The Ligand A1 was synthesized according to the literature method (Li et al., 2018). 3-Carboxylic acid*-*2H-Chromen-2-one (1 mmol, 190 mg) was dissolved in DCM. EDCI (1.1 mmol, 210 mg) and DMAP(0.15 mmol, 18 mg) were added to the mixture solution. The mixture was stirred at 0°C. for an hour. Propargylamine (1 mmol, 69 μL) was slowly added. The stirring was continued for 1 h at 0°C.and then at room temperature for 24 h until the reaction completed. The reaction mixture was then concentrated. The obtained crude product was purified by column chromatography 0.16 g white solid obtained with 70% yield. mp: 188–189°C. ^1^H NMR (400 MHz, DMSO-d6) δ 8.92 (t, J = 5.6 Hz, 1H), 8.84 (d, J = 2.4 Hz, 1H), 7.99–7.91 (m, 1H), 7.73 (tq, J = 8.6, 7.2, 2.0 Hz, 1H), 7.50–7.38 (m, 2H), 4.11 (dd, J = 4.8, 2.4 Hz, 2H), 3.15 (t, J = 2.4 Hz, 1H). 13C NMR (151 MHz, DMSO-d6) δ 166.41, 165.62, 159.33, 153.30, 139.66, 135.76, 130.57, 123.90, 123.79, 121.57, 86.14, 78.71, 34.17.

The preparation of complexes B1 was similar to A1.

7-Diethylamino-2-Oxo-N-2-propyn-1-yl-3-carboxamide-2H-Chromen-2-one (B1): A 0.193 g portion of yellow solid was isolated with yield 65%. mp: 195–196°C. ^1^H NMR (400 MHz, Chloroform-*d*) δ 8.99 (t, *J* = 5.2 Hz, 1H), 8.68 (s, 1H), 7.42 (d, *J* = 8.8 Hz, 1H), 6.64 (dd, *J* = 8.0, 2.5 Hz, 1H), 6.48 (d, *J* = 3.6 Hz, 1H), 4.23 (dd, *J* = 5.6, 2.8 Hz, 2H), 3.45 (q, *J* = 6.8 Hz, 4H), 2.25 (t, *J* = 2.4Hz, 1H), 1.24 (t, *J* = 7.2 Hz, 6H). ^13^C NMR (101 MHz, Chloroform-*d*) δ 163.18, 162.79, 157.92, 152.89, 148.58, 131.42, 110.22, 109.82, 108.48, 96.75, 80.01, 71.39, 45.32, 29.38, 12.63.

The Ligands C1-G1 was synthesized according to the literature method (Gong et al., 2015).

7-(2-Propyn-1-yloxy)-2H-Chromen-2-one (C1): A 0.162 g portion of white solid was isolated with yield 81.2%. mp: 124–125°C. ^1^H NMR (400 MHz, Chloroform-*d*) δ 7.69–7.61 (m, 1H), 7.38 (m, 1H), 6.89 (m, 2H), 6.25 (dt, *J* = 8.0, 2.0 Hz, 1H), 4.74 (t, *J* = 2.0 Hz, 2H), 2.57 (q, *J* = 2.0 Hz, 1H). ^13^C NMR (151 MHz, Chloroform-d) δ 160.94, 160.49, 155.58, 143.26, 128.82, 113.59, 113.15, 112.98, 102.09, 77.36, 76.55, 56.18.

7-(2-Propyn-1-yloxy)-3-Acetyl--2H-Chromen-2-one (D1): 0.153 g light yellow solid. Yield:63.4%. mp:183–184°C. 1H NMR (400 MHz, DMSO-d6) δ 8.60 (d, J = 0.8 Hz, 1H), 7.86 (dd, J = 8.8, 1.2 Hz, 1H), 7.07 (d, J = 2.4 Hz, 1H), 7.02 (dt, J = 8.8, 1.8 Hz, 1H), 4.96 (d, J = 2.4 Hz, 2H), 3.68–3.66 (m, 1H), 2.54 (s, 3H). 13C NMR (151 MHz, DMSO-d6) δ 195.17, 162.92, 159.18, 157.16, 147.87, 132.63, 121.31, 114.24, 112.74, 101.72, 79.61, 78.65, 56.86, 30.48.

7-(2-Propyn-1-yloxy)-4-methyl-2H-Chromen-2-one (E1): 0.114 g light yellow solid. Yield: 53.2%. mp: 143–145°C. ^1^H NMR (400 MHz, Chloroform-*d*) δ 7.54–7.49 (m, 1H), 6.92 (m, 2H), 6.14 (dq, *J* = 2.8, 1.2 Hz, 1H), 4.75 (t, *J* = 2.4 Hz, 2H), 2.57 (m, 1H), 2.39 (t, *J* = 1.2 Hz, 3H). ^13^C NMR (151 MHz, Chloroform-*d*) δ 161.06, 160.32, 155.00, 152.38, 125.59, 114.23, 112.67, 112.39, 102.14, 77.40, 76.47, 56.15, 18.64.

7-(2-Propyn-1-yloxy)-4-Difluoromethyl-2H-Chromen-2-one (F1): 0.067 g light yellow solid. Yield: 25.4%. mp: 120–121°C. 1H NMR (400 MHz, Chloroform-d) δ 7.64 (dt, J = 8.8, 1.2 Hz, 1H), 7.03–6.93 (m, 2H), 6.72 (td, J = 53.6, 0.8 Hz, 1H), 6.49 (dt, J = 1.6, 0.8 Hz, 1H), 4.78 (d, J = 2.4 Hz, 2H), 2.59 (s, 1H). 13C NMR (151 MHz, Chloroform-d) δ 160.93, 159.83, 155.95, 145.05, 125.90, 113.73, 113.46, 112.32, 112.26, 112.20, 112.12, 110.51, 109.05, 102.64, 56.24. 19F NMR (376 MHz, Chloroform-d) δ -118.63, -118.83.

7-(2-Propyn-1-yloxy)-4- trifluoromethyl-2H-Chromen-2-one (G1): 0.13 g white solid. Yield: 48.6%. mp: 114–116°C 1H NMR (400 MHz, Chloroform-d) δ 7.63 (m, 1H), 7.03–6.92 (m, 2H), 6.62 (t, J = 0.8 Hz, 1H), 4.78 (d, *J* = 3.0 Hz, 2H), 2.59 (t, *J* = 2.4 Hz, 1H). ^13^C NMR (101 MHz, DMSO-*d*_6_) δ 156.66, 154.66, 151.50, 121.89, 118.36, 115.62, 109.24, 108.33, 108.27, 108.21, 108.16, 103.11, 98.12, 72.86, 72.23, 51.76. ^19^F NMR (376 MHz, Chloroform-*d*) δ -65.17.

### Synthesis of the complexes 1-7

#### [2-Oxo-N-2-propyn-1-yl*-*3-carboxamide*-*2H-chromen-2-one] hexacarbonyl-dicobalt (1)

The complex 1 was synthesized according to the literature method (Gong et al., 2015). LIIn the presence of Ar atmosphere, 2-Oxo-*N*-2-propyn-1-yl*-*3-carboxamide*-*2H-Chromen-2-one (A1) (1 mmol, 227 mg) was dissolved in 15 ml of dry THF, and then Co_2_(CO)_8_ (1.2 mmol, 410.4 mg) was added. The mixture was stirred for 12 h at room temperature. The solvent was removed under reduced pressure. The crude was purified by silica gel column chromatography. A 180.5 mg portion of brown-red solid was isolated with yield 35.2%.mp: >250°C ^1^H NMR (400 MHz, Chloroform-*d*) δ 9.42–9.32 (m, 1H), 8.99 (s, 1H), 7.79–7.67 (m, 2H), 7.50–7.39 (m, 2H), 6.14 (s, 1H), 4.86 (d, *J* = 6.0 Hz, 2H). ^13^C NMR (151 MHz, Chloroform-*d*) δ 199.19, 161.49, 154.49, 148.72, 134.18, 129.84, 125.29, 118.58, 118.00, 116.69, 92.01, 72.19, 42.08. ESI-HRMS (m/z): calcd for C_19_H_9_Co_2_NaNO_9_ [M+Na]^+^: 535.8834; found 535.8835.

Complexes 2-7 were synthesized following the same procedure as for complex 1.

#### [7-Diethylamino-2-Oxo-N-2-propyn-1-yl*-*3-carboxamide*-*2H-chromen-2-one] hexacarbonyl-dicobalt (2)

A 149.5 mg portion of brown-red solid was isolated with yield 25.6%. mp: >250°C. ^1^H NMR (400 MHz, Chloroform-*d*) δ 9.31 (t, *J* = 6.0 Hz, 1H), 8.73 (s, 1H), 7.43 (dd, *J* = 9.0, 0.8 Hz, 1H), 6.65 (ddd, *J* = 9.0, 2.6, 0.8 Hz, 1H), 6.49 (d, *J* = 2.4 Hz, 1H), 6.09 (q, *J* = 1.0 Hz, 1H), 4.80 (dt, *J* = 6.0, 1.0 Hz, 2H), 3.45 (q, *J* = 7.2 Hz, 4H), 1.28–1.20 (m, 6H). ^13^C NMR (151 MHz, Chloroform-*d*) δ 199.34, 163.08, 162.60, 157.69, 152.60, 148.31, 131.16, 109.93, 109.79, 108.37, 96.61, 92.96, 72.20, 45.08, 41.79, 12.38. ESI-HRMS (m/z): calcd for C_23_H_19_Co_2_N_2_O_9_ [M+H]^+^: 584.9749; found 584.9745.

#### [7-(2-Propyn-1-yloxy)-2H-chromen-2-one] hexacarbonyl-dicobalt (3)

A 153.6 mg portion of brown-red was isolated with yield 33.5%. mp:142–145°C. ^1^H NMR (400 MHz, Acetone-*d*_6_) δ 7.92 (s, 1H), 7.74–7.45 (m, 1H), 7.01 (s, 2H), 6.59 (s, 1H), 6.25 (d, *J* = 7.2 Hz, 1H), 5.52 (s, 2H). ESI-HRMS (m/z): calcd for C_18_H_9_Co_2_NaO_9_ [M+H]^+^: 486.8905; found 486.8900.

#### [7-(2-Propyn-1-yloxy)-3-acetyl--2H-Chromen-2-one] hexacarbonyl-dicobalt (4)

A 160.5 mg portion of brown-red solid was isolated with yield 30.4%. mp:153–156°C.^1^H NMR (400 MHz, Acetone-*d*_6_) δ 8.54 (s, 1H), 7.87 (s, 1H), 7.07 (s, 2H), 6.61 (s, 1H), 5.59 (s, 2H), 2.59 (s, 3H). ESI-HRMS (m/z): calcd for C_20_H_10_Co_2_NaO_10_ [M+Na]^+^: 550.8830; found 550.8831.

#### [7-(2-Propyn-1-yloxy)-4-methyl-2H-chromen-2-one] hexacarbonyl-dicobalt (5)

A 142.9 mg portion of brown-red solid was isolated with yield 28.6%. mp:163–165°C. ^1^H NMR (400 MHz, Acetone-*d*_6_) δ 7.73 (d, *J* = 8.8 Hz, 1H), 7.08–6.93 (m, 2H), 6.59 (s, 1H), 6.15 (s, 1H), 5.52 (s, 2H), 2.45 (s, 3H). ^13^C NMR (101 MHz, Acetone-*d*_6_) δ 199.87, 161.54, 160.14, 155.59, 152.98, 126.54, 114.08, 112.62, 111.98, 101.73, 89.74, 72.72, 69.10, 17.90. ESI-HRMS (m/z): calcd for C_19_H_10_Co_2_NaO_9_ [M+Na]^+^: 522.8881; found 522.8887.

#### [7-(2-Propyn-1-yloxy)-4*-*difluoromethyl-2H*-*chromen-2-one] hexacarbonyl-dicobalt (6)

A 116.8 mg portion of brown-red solid was isolated with yield 21.8%. mp:135–137°C.^1^H NMR (400 MHz, Acetone-*d*_6_) δ 7.81 (d, *J* = 8.4 Hz, 1H), 7.37–7.08 (m, 2H), 7.07 (d, *J* = 2.8 Hz, 1H), 6.57 (d, *J* = 17.2 Hz, 2H), 5.55 (s, 2H). ^19^F NMR (376 MHz, Acetone-*d*_6_) δ -120.07, -120.21. ESI-HRMS (m/z): calcd for C_19_H_9_Co_2_F_2_O_9_ [M+H]^+^: 536.8873; found 536.8866.

#### [7-(2-Propyn-1-yloxy)-4*-* trifluoromethyl-2H-chromen-2-one] hexacarbonyl-dicobal (7)

A 137 mg portion of brown-red oil was isolated with yield 24.7%. mp:101–103°C. ^1^H NMR (400 MHz, Acetone-*d*_6_) δ 7.91–7.73 (m, 1H), 7.22 (d, *J* = 9.6 Hz, 2H), 6.82 (s, 1H), 6.69 (s, 1H), 5.66 (s, 2H). ^13^C NMR (101 MHz, Acetone-*d*_6_) δ 205.01, 167.59, 163.87, 161.85, 145.93, 145.61, 131.70, 128.76, 126.03, 119.05, 118.58, 118.53, 118.47, 118.42, 112.52, 107.68, 94.42, 77.91, 74.63. ^19^F NMR (376 MHz, Acetone-*d*_6_) δ -65.30. ESI-HRMS (m/z): calcd for C_19_H_8_Co_2_F_3_O_9_ [M+H]^+^: 554.8779; found 554.8753.

### Details of crystallographic analysis

Diffraction data were collected on a SuperNova Dual Cu/Mo diffractometer fitted with Mo-Kα radiation (λ = 0.71070 Å). The crystal was kept at 293.19(12) K during data collection. Using Olex2 (Dolomanov et al., 2009), the structure was solved by direct methods with the SHELXS97 (Sheldrick, 2008) program and refined with SHELXL97 by full-matrix least-squares method. CCDC 2063062 (5) contain the supplementary crystallographic data for this paper.

## Results and discussion

### Drug design, synthesis, characterization and properties

**SCHEME 1:**
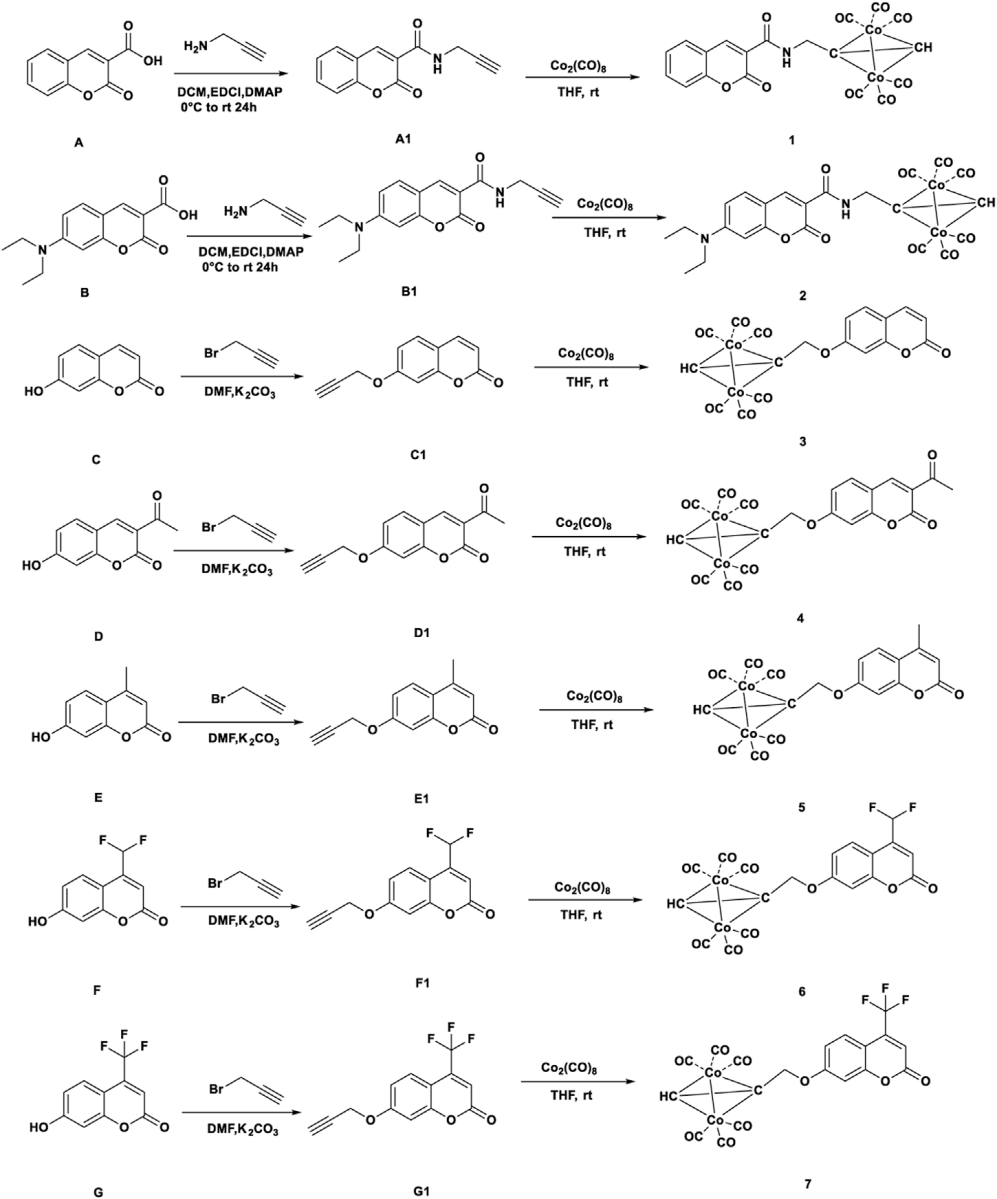
Syntheses and structures of Complexes 1–7.

Compound A was obtained by refluxing salicylaldehyde with meldrum’s acid in water. Compound B was prepared by reacting 4-(diethylamino)-2-hydroxybenzaldehyde, meldrum’s acid, piperidine with acetic acid in ethanol. Compound E, F and G were synthesized by the reaction of resorcinol with ethyl acetoacetate or substituted ethyl acetoacetate in the presence of concentrated sulfuric acid for 20 h. Then ligands A1-G1 reacted with Co_2_(CO)_8_ to form complexes 1–7. The structures and synthetic routes for complexes 1-7 were shown in Scheme 1. All the complexes were red-black oil or red solid. All cobalt complexes 1-7 were poor water-solubility and could dissolve in organic solvents, such as DMSO, THF, acetone, methanol.

**FIGURE 1:**
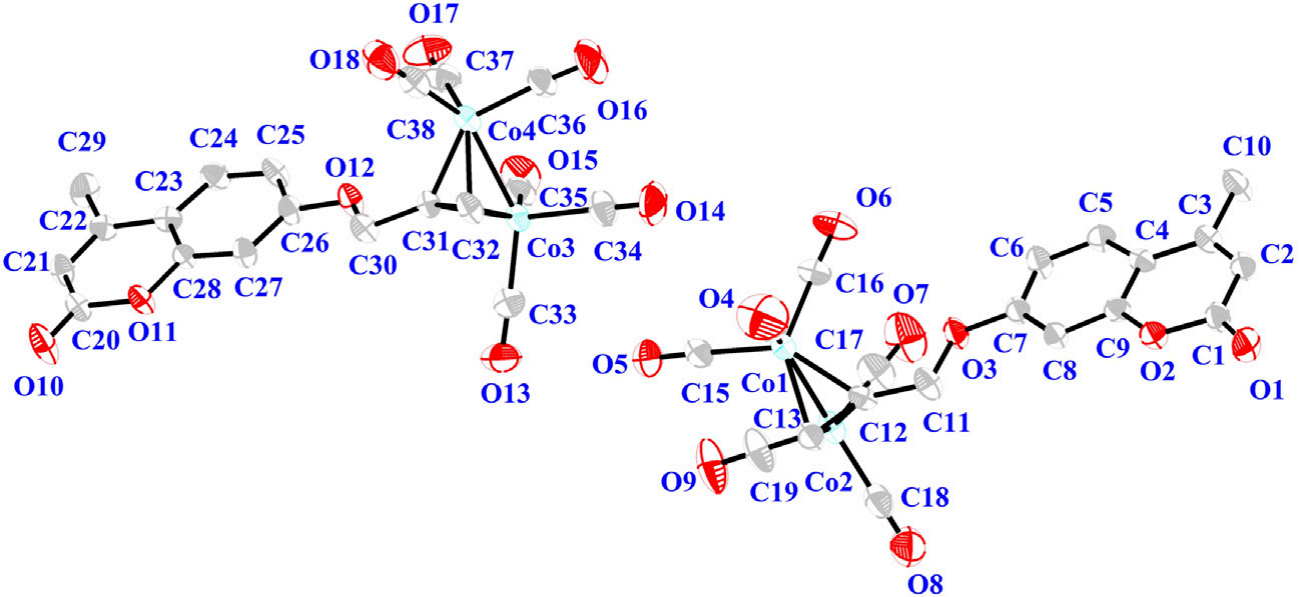
Thermal ellipsoid plot of the cation of complex 5 shown with 30% probability ellipsoids. H atoms and the counterion are omitted for the sake of clarity.

In addition, 5 were determined by single-crystal X-ray diffraction, their molecular structures were shown in Figure 1. Details of the data collection and structure refinement were in SI-Supplementary Table S1. The partial bond length and bond angle of complex 5 were shown in SI-Supplementary Table S2. Complex 5 had a twisted tetrahedral structure. The monoclinic Centrosymmetric lattice of complex 5 contained two molecules in the cell. Each Co atom in the molecule was connected with three carbonyl groups, in which the bond length of Co-Co was 2.473 (2) and 2.4615 (19), respectively. Six cobalt-carbon bonds were formed between two cobalt atoms and six carbonyl carbons. The bond lengths of the six cobalt-carbon bonds were different, which were in the range of 1.768 (11) ∼ 1.961 (8). The bond length of cobalt-carbon bond was longer than that of other bonds in the complex 5.

### Anti-tumor activities of the complexes

The anti-proliferation activities of the novel synthesized complexes on the Hela, MDAMB231 and HepG2 cells line were evaluated by MTT. The cells were incubated with complexes at different concentrations 10–150 μM for 24 h. The results were shown in Table 1. Through the study of structure-activity relationship, it was found that the substituted of C4-position electron donor groups on the pyran ring could remarkably influence the activity. Complexes 5 exhibited high activity against all three cancer cell lines. The IC_50_ values of complex 5 against all cancer cell lines were similar to or better than the anticancer drug 5-Fu. Introducing the electron withdrawing group on the pyran ring caused slight reduction of the activity. For the complexes 6, IC_50_ values were 145.26 μM, 153.39 μM and 547.06 μM against Hela, HepG2 and MDAMB231 cancer cells respectively. Two complexes 6 and 7 were lower cytotoxicity against MDAMB231 cells than complex 5. It was unfortunately that complexes 6 and 7 containing F atom were almost inactive. Complex 1 showed better antitumor activity than complex 2 against HepG2. Complexes 1 and 3 exhibited a moderate cytotoxicity against the HepG2 cancer cell lines. The rest complexes showed moderate activity. From these results, we concluded that compound substituted with electron donating methyl group on pyran ring showed good activity, which revealed that substitution on pyran ring played a vital role in the activity.

**TABLE 1:** IC50 values of all the complexes to the tumor cells. The results were represented as average values ± standard deviation. Data represent at least three independent experiments.

Complex	IC50 (μM)a		
	Hela	HepG2	MDA-MB-231
1	223.66 ± 49.43	54.04 ± 2.67	667.35 ± 279.95
2	84.34 ± 14.08	117.56 ± 16.48	210.32 ± 121.90
3	73.59 ± 4.42	50.60 ± 1.56	116.64 ± 6.02
4	105.11 ± 5.31	81.44 ± 3.04	59.69 ± 12.58
5	93.68 ± 21.40	48.15 ± 4.58	34.98 ± 7.57
6	145.26 ± 6.61	153.39 ± 6.31	547.06 ± 175.60
7	377.60 ± 79.73	131.31 ± 19.62	109.47 ± 13.13
5-FU	115.87 ± 6.44	205.25 ± 18.69	83.04 ± 2.99

### The stability of complex 5

**FIGURE 2:**
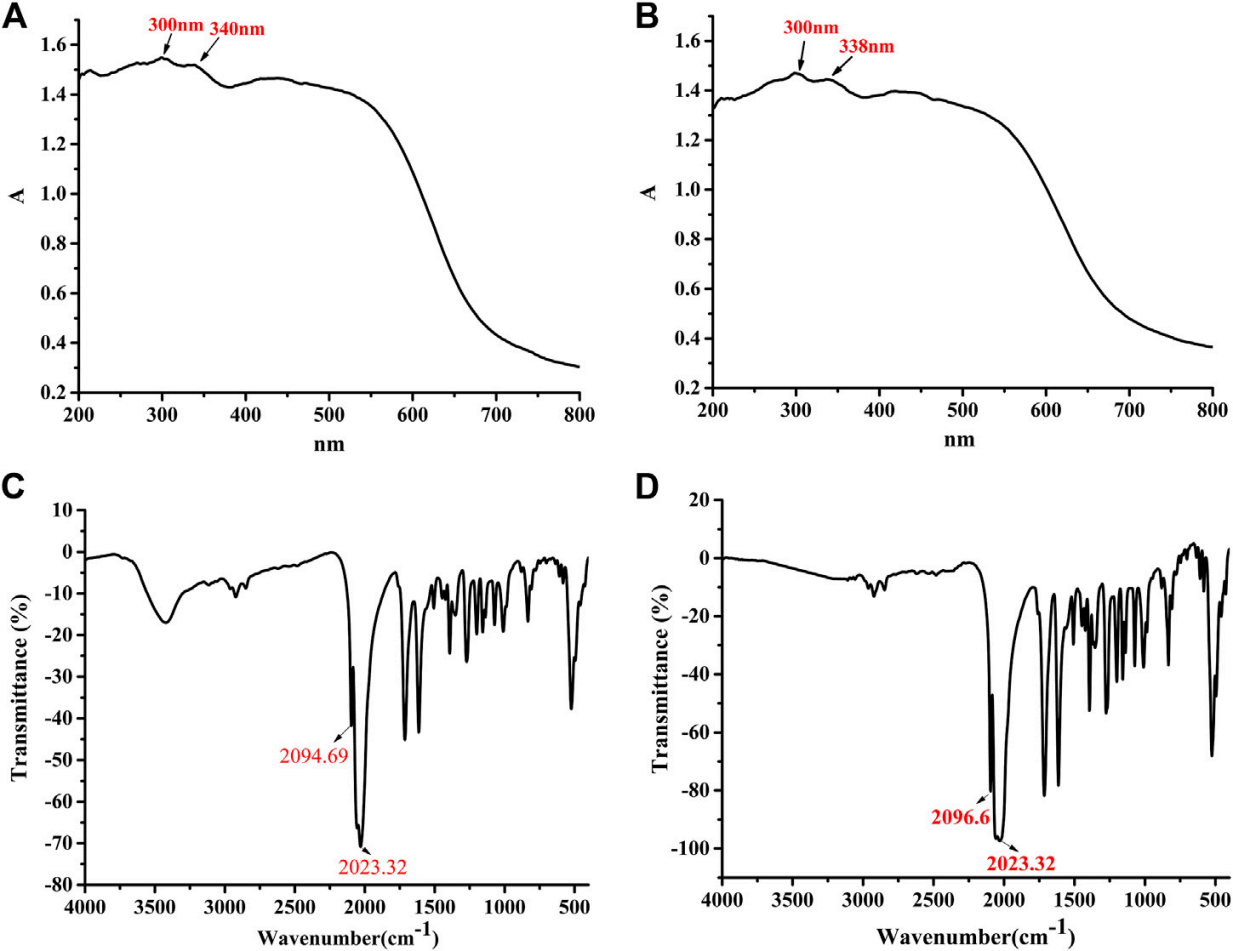
The UV spectrum of complex 5 after exposed to the air for 7 d. [0 d **(A)**, 7 d **(B)**]. The Infrared spectrum of complex 5 after exposed to the air for 7 d. [0 d **(C)**, 7 d **(D)**].

**FIGURE 3:**
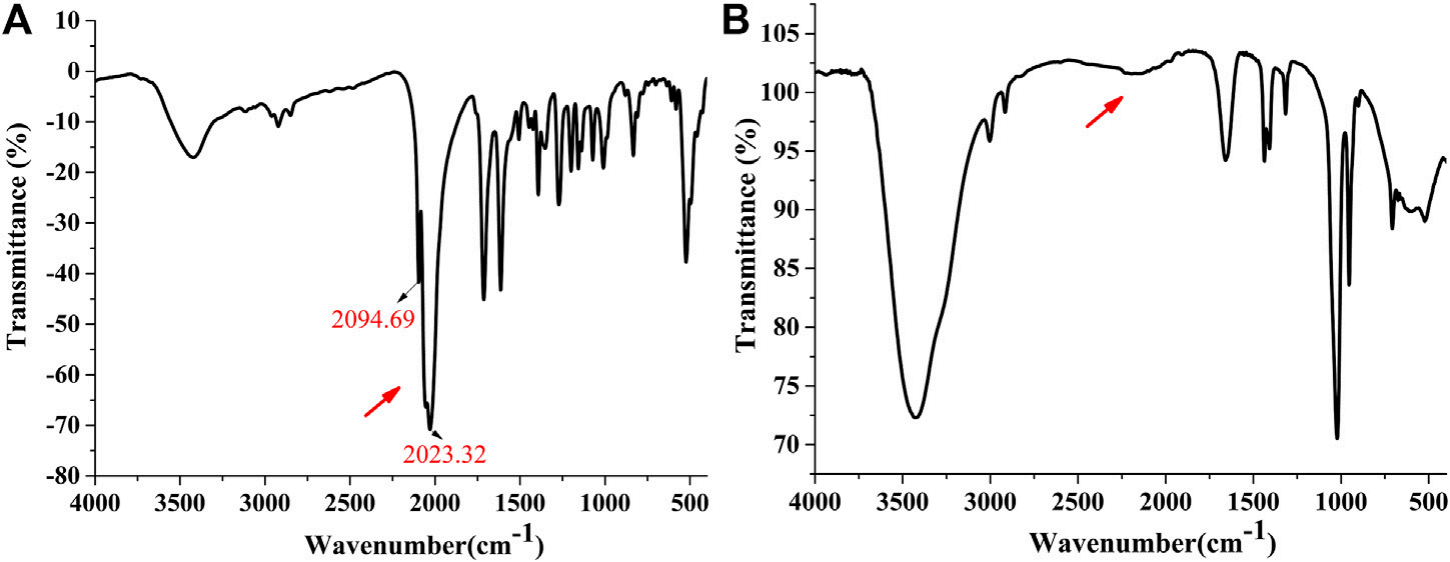
The infrared spectrum of complex 5 [The solid powder **(A)**; incubated at 37°C, 0.1 M PBS, pH = 7.4 for 24 h **(B)**].

The solid sample of complex 5 was further exposed to air. After 7 days, the UV diffuse reflectance spectrum exhibited that the absorbance was not changed slightly near the maximum absorption wavelength 300 nm (see Figure 3). When complex 5 was placed in the air for 7 days, infrared spectroscopy results showed that there were metal carbonyl groups at 2023–2096 cm^−1^, indicating that compound 5 was rather stable and not released carbon monoxide in solid state (see Figure 2). It was further demonstrated that compound 5 could be used as an effective carrier for CO.

In order to study whether complex 5 can release CO molecules, we investigated the release behavior of complex under physiological conditions. The complex 5 was incubated at 37°C, 0.1 M PBS and pH = 7.4 for 24 h (physiological conditions). Compared with the infrared spectra of solid powder, the infrared spectra showed that the metal carbonyl peak of complex 5 disappeared, indicating that complex 5 could release CO under physiological conditions (see Figure 3, the solid powder(a); incubated at 37°C, 0.1 M PBS, pH = 7.4 for 24 h(b). It was further demonstrated that complex 5 (CORM) could not only be used as an effective carrier for CO but also release the CO under physiological conditions.

### Cell viability

**FIGURE 4:**
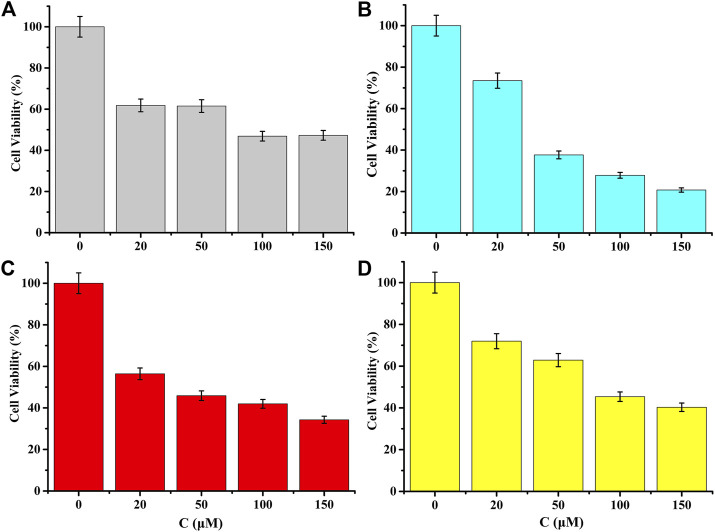
MTT assays on tumor cell lines showing cell viability (for **(A)** Hela of complex 5, for **(B)** HepG2 of complex 5, for **(C)** MDA-MB-231 of complex 5 and for **(D)** MDA-MB-231 of 5-FU). Data represent at least three independent experiments.

Cell viabilities were measured using the MTT assay as noted. Cell viability could be used to show the effects of complexes on the growth of different tumor cells. When complex 5 was used to act on tumor cells at a concentration of 20 μM, the cell viability of HepG2, HeLa and MDA-MB-231 tumor cells were 73.47%, 61.8%, and 56.40%, respectively. When the tumor cells were treated with 50 μM, the cell survival rates of HepG2, HeLa and MDA-MB-231 tumor cells were 37.67%, 61.49%, and 45.89%, respectively. Compound 5 had a stronger inhibitory effect on the growth of MDA-MB-231 cells. Further, the complex 5 had considerable selectivity among the three kinds of tumor cells (see Figure 4).

### ROS

Duing to the ROS level in cancer cells higher than the normal cells, cancer cells were more vulnerable to further oxidative stress than normal cells. The vulnerability of cancer cells to oxidative signals was deemed to be a potential target for the exploited new anticancer drugs (Montero and Jassem, 2011; Ray et al., 2012). Mitochondrial ROS (superoxide free radical) was considered to be an important signal molecule. High levels of mitochondrial ROS could promote the apoptosis of tumor cells (Vyas et al., 2016; Sies and Jones, 2020).

**FIGURE 5:**
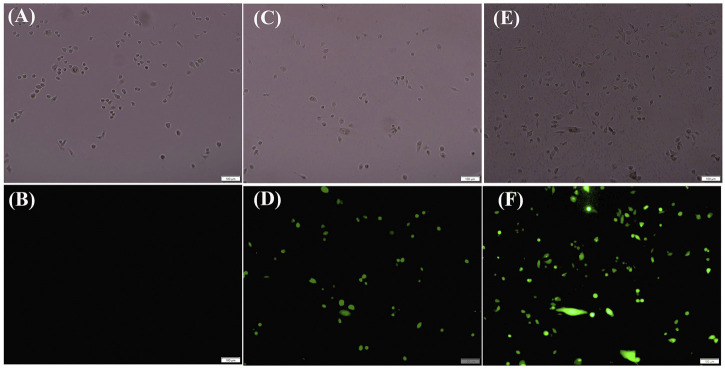
The production of ROS of Tumor Cells MDAMB231 treated with 5 visualized by fluorescence microscope images of MDAMB231 cells. Control **(A,B)**, for 40 μM **(C,D)**, 80 μM **(E,F)** after treated with complex 5 for 24 h.

MDAMB231 cells were treated with compound 5 of different concentrations (40 μM, 80 μM) for 24 h. After stained with ROS, DCFH-DA was rapidly oxidized into fluorescence dichlorofluorescein (DCF). The results showed that different concentrations (40 μM, 80 μM) on these cells induced significantly higher ROS generation. Compared with the untreated negative control cells, the results suggested that the experimental group exhibited significant accumulation of reactive oxygen species (see Figure 5). Coumarin derivatives were Self-fluorescence. But the Co-CORM conjugated coumarin derivatives was not contributed to fluorescence. The Co-CORM could make the fluorescence quenching of coumarin derivatives (see SI-Supplementary Figure S1).

### Mitochondrial membrane potential

**FIGURE 6:**
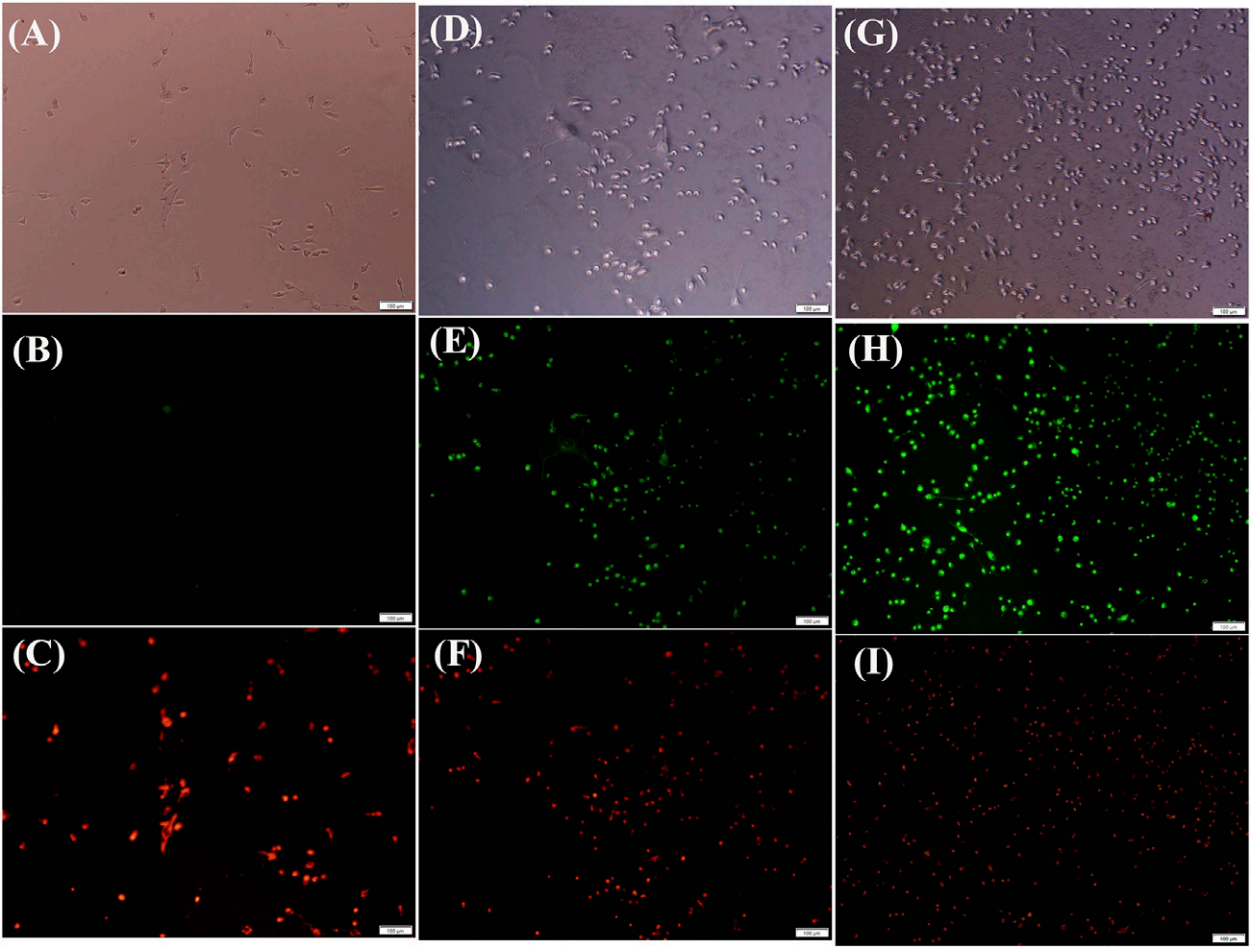
MMP was detected by JC-1 dye staining visualized with a fluorescence microscope. Control **(A,B,C)**, for 40 μM **(D,E,F)**, 80 μM **(G,H,I)** after treated with complex 5 for 24 h.

To determine whether the mitochondrial membrane potential (∆Ψ m) (MMP) is involved in the process of CORM-induced cell apoptosis, MDAMB231 cells were treated with compound 5 for 24 h. JC-1 was used to detect the mitochondrial membrane potential according to the manufacturer’s instructions. Compared with the control group, the green fluorescence intensity increased, while the red fluorescence decreased with the increase of the concentration of complex 5 in MDAMB231 cells. The mitochondrial membrane was depolymerized into singlet state (see Figure 6). These finding revealed that complex 5 could induce MMP consumption in MDAMB231 cells in a dose-dependent manner. Similarly, the CORM conjugated coumarin derivatives was also not contributed to fluorescence (see SI-Supplementary Figure S2). The results suggested that complex 5 might activate the mitochondrial-mediated apoptosis pathway in the MDAMB231 cells (see Figure 6).

### Western blotting

**FIGURE 7:**
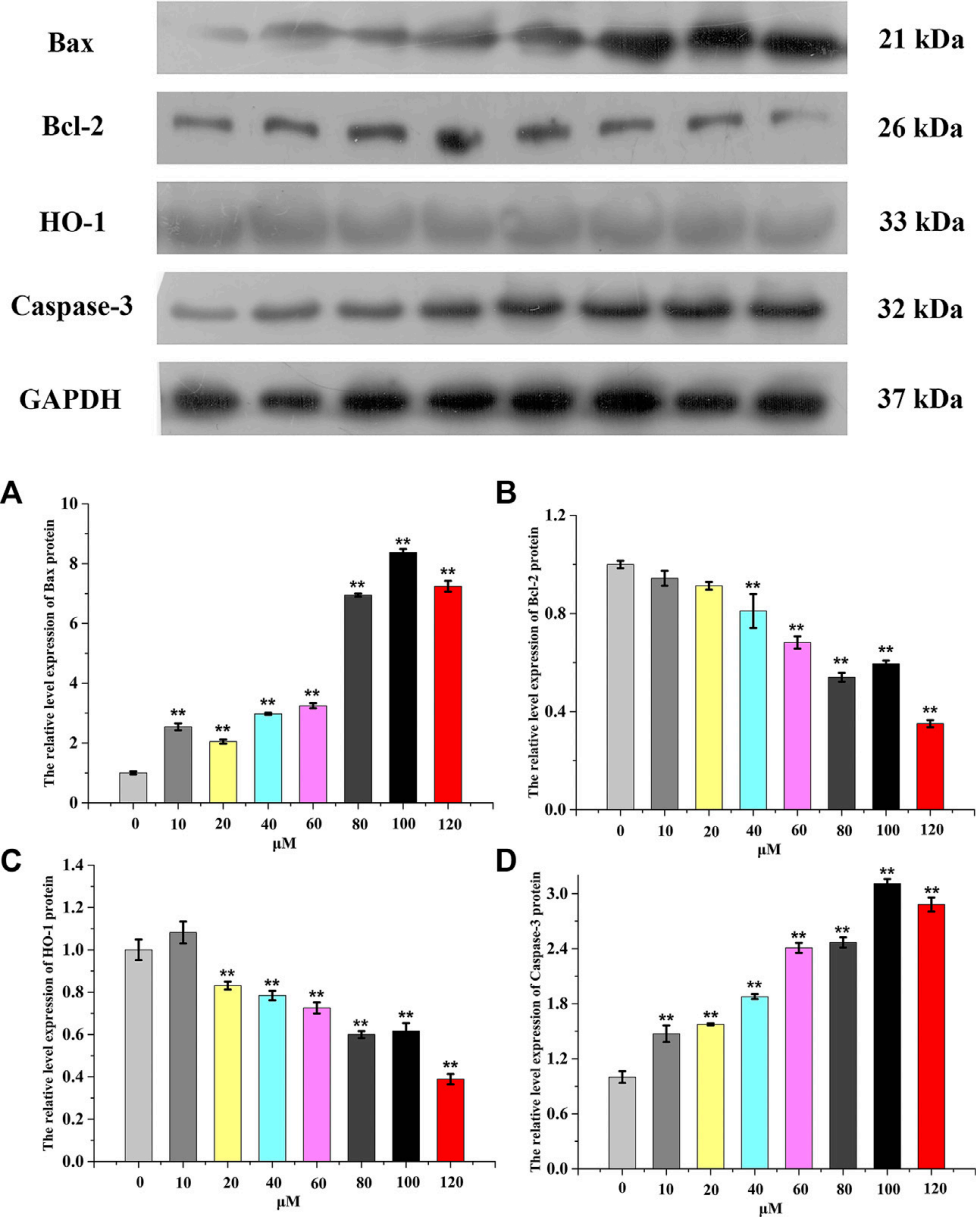
Changes in Bax **(A)**, Bcl-2 **(B)**, HO-1 **(C)** and Caspase-3**(D)** cell expression treated with complex 5 against HepG2 cells for 24 h. GAPDH was used as the loading control. Data are presented as the mean ± SEM. *: *p* < 0.05, **: *p* < 0.01. Each concentration compared to the control.

It was well known that caspase 3 affected the process of apoptosis, which led to cell death through the proteolysis of a large number of cellular components and the activation of pro-apoptotic factors (Javid et al., 2011; Wang et al., 2019). HO-1 (heme oxygenase) could catalyze heme to produce bilirubin and biliverdin, which was a potential antioxidant (Stocker et al., 1990). Bcl-2 protein was mainly located in mitochondria, which was the key determining point (Wang et al., 2019). In many drug-resistant tumor cells, the expression level of Bcl-2 was increased (Sakamoto and Kyprianou, 2010). MDAMB231 cells were treated with complex 5 at certain concentrations (10 μM–120 μM). Western blotting showed that complex 5 could up-regulate the expression of Bax protein, down-regulate the expression of Bcl-2 and activate caspase-3, down-regulate the expression of HO-1 in different concentrations compared with the control (see Figure 7). Therefore, the apoptosis induction of complex 5 in MDAMB231 cells was related to the mitochondrial dysfunction signal pathway.

### Molecular docking simulation

**FIGURE 8:**
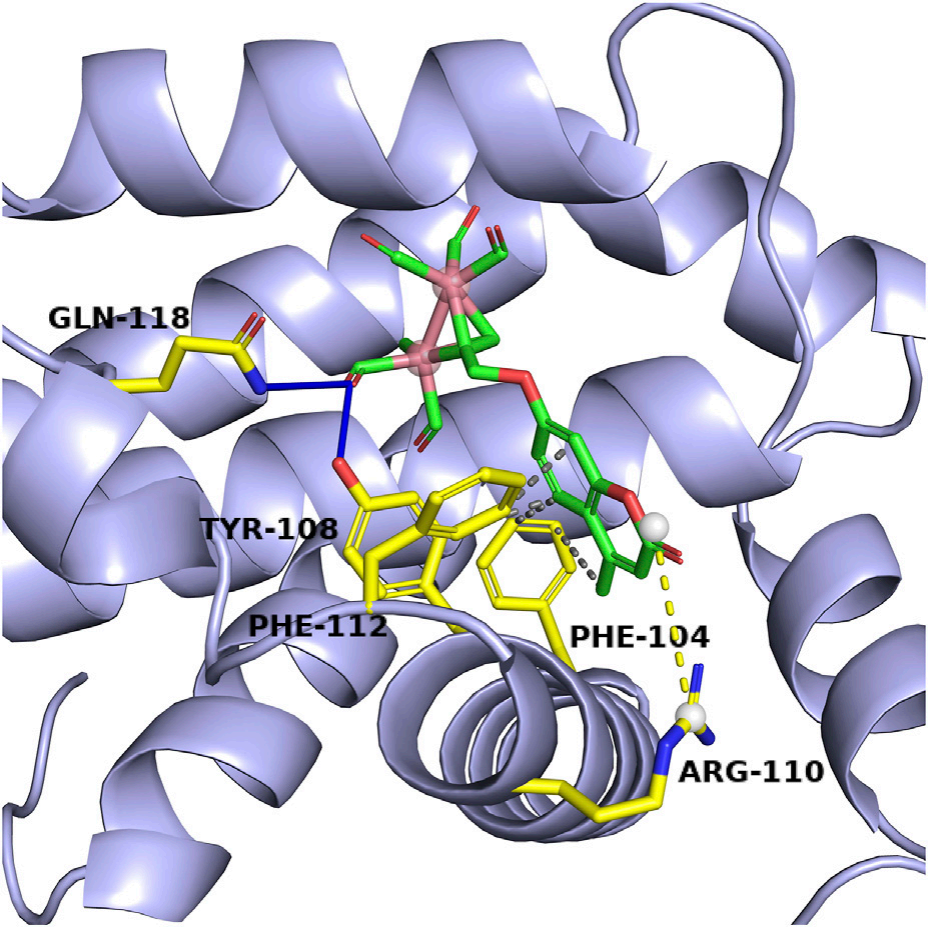
Schematic representation of the interactions between the complex 5 and the Bcl-2 protein. The blue dashed line is the hydrogen bond interaction, the grey dashed line is the hydrophobic interaction, the yellow dashed line is the salt bridge, the yellow sticks model is the amino acid residue and the green sticks model is the target small molecule.

The binding mode of Complex 5 to Bcl-2 were established by vina autodock tool. In the optimal binding conformation, the binding free energy of the target small molecule to the target protein Bcl-2 was -6.3 kcal/mol. Compound 5 had hydrophobic interaction with amino acid residues PHE104, TYR108 and PHE112 of Bcl-2. These forces support the stable existence of complex 5 in the Bcl-2 cavity. The amino acid TYR108 and GLN118 are connected to compex 5 through hydrogen bond. Additional, the complex 5 also is directed with ARG110 through salt bridge interaction. The results suggest that the salt bridge formation would also be involved in the stronger interaction between the Bcl-2 and the complex 5 (see Figure 8) (see Tables 2–4). Therefore, it can be concluded that salt bridge, hydrophobic interaction and hydrogen bonding play a vital role in maintaining the stability of the Bcl-2/complex.

**TABLE 2:** The hydrophobic interaction between Complex 5 and Bcl-2.

Hydrophobic interactions				
Index	Residue	Distance	Ligand Atom	Protein Atom
1	PHE104	3.83	1165	301
2	PHE104	3.27	1158	303
3	TYR108	3.53	1158	339
4	PHE112	3.69	1162	382

**TABLE 3:** The hydrogen bonds between Complex 5 and Bcl-2.

Hydrogen bonds						
Index	Residue	Distance H-A	Distance D-A	Donor angle	Donor atom	Acceptor atom
1	TYR108	2.16	2.93	138.27	343 [O3]	1169 [O2]
2	GLN118	3.54	3.93	106.04	427 [Nam]	1169 [O2]

**TABLE 4:** The Salt bridges between Complex 5 and Bcl-2.

Salt bridges				
Index	Residue	Distance	Ligand Group	Ligand Atoms
1	ARG110	5.23	Carboxylate	1173, 1174

### Pharmacokinetic properties

The estimation of druglike and ADME parameters (Log *P*_o/w_, LogK_P_, BBB, TPSA et al.) were operated by using Swiss ADME tool. Polar surface area was a key determinant of fractional absorption (Jin et al., 2018). Coumarin derivatives might be metabolized mainly by liver microsomal P450 enzymes (Mäenpää et al., 1993). The values of TPSA of all complexes were less than 140 Å^2^, indicating that all the predicted complexes had good cell membrane permeability and transdermal absorption ability. The rotational bonds showed that the synthesized complexes had good intestinal absorption capacity. Absorption and distribution properties of the synthesized complexes were shown in Table 5. The metabolism of complex 3 with 7-hydroxycoumarin as ligand was different from that of complex 5, 6 and 7, indicating that the modification of substituents at position 4 would change the metabolic mode of drugs. Complex 1-7 might inhibit CYP2C9 and could be used as substrates of P-gp (see Table 6). The bioavailability refer to Probability of F>10% in rat implement from the reference (Martin, 2005). The acute toxicity studies (LD_50_) was calculated by ProTox-II server (https://tox-new.charite.de/) (Banerjee et al., 2018). The values indicate the complexes for which side effects are less likely (see Table 7). The Lipinski’s rule is to evaluate whether a compound can be used as a drug, or a compound with pharmacological activity or druglike properties. Lipinski´s rule of 5 which required: molecular weight MWt ≤ 500 g/mol, number of hydrogen bond donor HBD ≤5, number of hydrogen bond acceptor HBA ≤ 10, number of rotatable bonds ≤ 10, and TPSA <140 Å2 and other properties (Żołek and Maciejewska, 2017). The synthetic complexes except 2 are in line with the Lipinski rule of five, which has good permeability and drug-like properties. The pharmacokinetics properties prediction revealed that the newly designed complexes except 2, obeyed the Lipinski´s rule for oral bio-availability.

**TABLE 5:** Absorption and distribution properties of the synthesized complexes.

Comp.	MW	Consensus Log Po/w	HBA	HBD	TPSA (≤ 140 (Å)	LogKP (skin permeation/cm/s)	Rotatable bond	BBB permeant
1	513.14	-0.32	9	1	59.312	−5.23	4	Yes
2	584.26	0.21	9	1	62.552	−5.06	7	Yes
3	486.12	-0.09	9	0	39.442	−5.11	3	Yes
4	528.15	-0.05	10	0	56.512	−5.45	4	Yes
5	500.14	0.11	9	0	39.442	−5.25	3	Yes
6	536.12	0.58	11	0	39.442	−5.3	4	Yes
7	554.12	0.83	12	0	39.442	−5.22	4	Yes

**TABLE 6:** Metabolism of all synthesized complexes *via* hepatic microsomal isoforms.

Sample	CYP1A2 inhibitor	CYP2C19 inhibitor	CYP2C9 inhibitor	CYP2D6 inhibitor	CYP3A4 inhibitor	P-gp substrate
1	No	No	Yes	No	No	Yes
2	No	Yes	Yes	No	Yes	Yes
3	No	No	Yes	No	No	Yes
4	No	No	Yes	No	No	Yes
5	No	Yes	Yes	No	Yes	Yes
6	No	Yes	Yes	No	Yes	Yes
7	No	Yes	Yes	No	Yes	Yes

**TABLE 7:** The bio-availability, Druglikeness and Toxicity properties of the synthesized complexes. Toxicity evaluation result (LD50). Evaluation processed by ProTox-II server (https://tox-new.charite.de/).

Comp.	Lipinski	Ghose	WLOGP	XLOGP3	Veber	Egan	Muegge	Bioavailability scores	LD50 (mg/kg)
1	Yes	NO	0.88	5.92	Yes	Yes	NO	0.55	906
2	NO	NO	1.73	6.77	Yes	Yes	NO	0.17	906
3	Yes	NO	1.53	5.85	Yes	Yes	NO	0.55	3200
4	Yes	NO	1.73	5.74	Yes	Yes	NO	0.55	5000
5	Yes	NO	1.84	5.77	Yes	Yes	NO	0.55	1100
6	Yes	NO	2.98	6.02	Yes	Yes	NO	0.55	1100
7	Yes	NO	3.7	6.28	Yes	Yes	NO	0.55	1100

## Conclusion

In conclusion, a series of novel carbonyl metal CORMs containing cobalt were designed and synthesized. The anticancer activity of the complexes was evaluated *in vitro* against MDAMB231, Hela and HepG2 cancer cell lines. All the IC_50_ values of complexes were in range of 34.98–667.35 μM. Among all the complexes, complex 5 was the most toxic, and its IC_50_ value was 34.98 μM. Complex 5 had the highest activity against HepG2 and MDAMB231 cancer cell line. More importantly, complex 5 obviously caused dissipation of MMP and effectively induced ROS production. Complex 5 up-regulated the expression of Bax protein and down-regulate the expression of Bcl-2 and HO-1, activate Caspase-3. The docking studies showed that complex 5 could be inserted into the active pocket of Bcl-2. It can be concluded that salt bridge, hydrophobic interaction and hydrogen bonding play a vital role in enhancing the binding affinity of the Bcl-2/complex. The results indicated that these complexes were promising candidates as anticancer therapeutic agents. Carbonyl cobalt carbon monoxide release molecules based on coumarin derivatives are expected to become a new trend for the design of anticancer drugs.

## Data Availability

The datasets presented in this study can be found in online repositories. The names of the repository/repositories and accession number(s) can be found in the article/Supplementary Material.
