# Supplementary material for: Synthesis, docking studies, biological activity of carbon monoxide release molecules based on coumarin derivatives
Source: Front Chem. 2022 Sep 29;10:996079. doi: 10.3389/fchem.2022.996079 (PMC9557063; doi:10.3389/fchem.2022.996079)

checkCIF/PLATON report

Structure factors have been supplied for datablock(s) liuhp_1106_3

THIS REPORT IS FOR GUIDANCE ONLY. IF USED AS PART OF A REVIEW PROCEDURE FOR PUBLICATION, IT SHOULD NOT REPLACE THE EXPERTISE OF AN EXPERIENCED CRYSTALLOGRAPHIC REFEREE.

No syntax errors found.   CIF dictionary   Interpreting this report

Datablock: liuhp_1106_3

```
Bond precision:  C-C = 0.0114 A          Wavelength=1.54184

Cell:            a=19.8707(9)    b=7.3081(3)        c=29.0760(14)
                 alpha=90        beta=106.176(5)    gamma=90
Temperature:     293 K

                 Calculated                 Reported
Volume           4055.2(3)                  4055.1(3)
Space group      P 21/c                     P 1 21/c 1
Hall group       -P 2ybc                    -P 2ybc
Moiety formula   C19 H10 Co2 O9             C19 H10 Co2 O9
Sum formula      C19 H10 Co2 O9             C19 H10 Co2 O9
Mr               500.13                     500.13
Dx,g cm-3        1.638                      1.638
Z                8                          8
Mu (mm-1)        13.267                     13.267
F000             2000.0                     2000.0
F000’            1969.07
h,k,lmax         24,8,35                    23,8,35
Nref             7679                       7481
Tmin,Tmax        0.764,0.876                0.314,1.000
Tmin’            0.376

Correction method= # Reported T Limits: Tmin=0.314 Tmax=1.000
AbsCorr = MULTI-SCAN

Data completeness= 0.974          Theta(max)= 70.058

R(reflections)= 0.0806( 3819)     wR2(reflections)= 0.1870( 7481)

S = 1.081                   Npar= 543
```

The following ALERTS were generated. Each ALERT has the format
test-name_ALERT_alert-type_alert-level.
Click on the hyperlinks for more details of the test.

Alert level C

```
PLAT220_ALERT_2_C NonSolvent   Resd 1  O   Ueq(max)/Ueq(min) Range        3.1 Ratio
PLAT220_ALERT_2_C NonSolvent   Resd 2  O   Ueq(max)/Ueq(min) Range        3.2 Ratio
PLAT234_ALERT_4_C Large Hirshfeld Difference C25      --C26      .       0.16 Ang.
PLAT242_ALERT_2_C Low    'MainMol' Ueq as Compared to Neighbors of        C18 Check
PLAT242_ALERT_2_C Low    'MainMol' Ueq as Compared to Neighbors of        C36 Check
PLAT341_ALERT_3_C Low Bond Precision on  C-C Bonds ...............    0.01138 Ang.
PLAT906_ALERT_3_C Large K Value in the Analysis of Variance ......     11.191 Check
PLAT906_ALERT_3_C Large K Value in the Analysis of Variance ......      3.583 Check
PLAT906_ALERT_3_C Large K Value in the Analysis of Variance ......      2.016 Check
PLAT911_ALERT_3_C Missing FCF Refl Between Thmin & STh/L=    0.600         49 Report
```

Alert level G

```
PLAT199_ALERT_1_G Reported _cell_measurement_temperature ..... (K)        293 Check
PLAT200_ALERT_1_G Reported   _diffrn_ambient_temperature ..... (K)        293 Check
PLAT343_ALERT_2_G Unusual sp?      Angle Range in Main Residue for        C12 Check
PLAT343_ALERT_2_G Unusual sp?      Angle Range in Main Residue for        C13 Check
PLAT343_ALERT_2_G Unusual sp?      Angle Range in Main Residue for        C31 Check
PLAT343_ALERT_2_G Unusual sp?      Angle Range in Main Residue for        C32 Check
PLAT794_ALERT_5_G Tentative Bond Valency for Co1       (III)     .       2.79 Info
PLAT794_ALERT_5_G Tentative Bond Valency for Co2       (III)     .       2.84 Info
PLAT794_ALERT_5_G Tentative Bond Valency for Co3       (III)     .       2.72 Info
PLAT794_ALERT_5_G Tentative Bond Valency for Co4       (III)     .       2.80 Info
PLAT910_ALERT_3_G Missing # of FCF Reflection(s) Below Theta(Min).          3 Note
PLAT912_ALERT_4_G Missing # of FCF Reflections Above STh/L=  0.600        136 Note
PLAT933_ALERT_2_G Number of OMIT Records in Embedded .res File ...          3 Note
PLAT941_ALERT_3_G Average HKL Measurement Multiplicity ...........        1.9 Low
PLAT978_ALERT_2_G Number C-C Bonds with Positive Residual Density.          0 Info
```

```
   0 ALERT level A = Most likely a serious problem - resolve or explain
   0 ALERT level B = A potentially serious problem, consider carefully
  10 ALERT level C = Check. Ensure it is not caused by an omission or oversight
  15 ALERT level G = General information/check it is not something unexpected

   2 ALERT type 1 CIF construction/syntax error, inconsistent or missing data
  10 ALERT type 2 Indicator that the structure model may be wrong or deficient
   7 ALERT type 3 Indicator that the structure quality may be low
   2 ALERT type 4 Improvement, methodology, query or suggestion
   4 ALERT type 5 Informative message, check
```

It is advisable to attempt to resolve as many as possible of the alerts in all categories. Often the minor alerts point to easily fixed oversights, errors and omissions in your CIF or refinement strategy, so attention to these fine details can be worthwhile. In order to resolve some of the more serious problems it may be necessary to carry out additional measurements or structure refinements. However, the purpose of your study may justify the reported deviations and the more serious of these should normally be commented upon in the discussion or experimental section of a paper or in the "special_details" fields of the CIF. checkCIF was carefully designed to identify outliers and unusual parameters, but every test has its limitations and alerts that are not important in a particular case may appear. Conversely, the absence of alerts does not guarantee there are no aspects of the results needing attention. It is up to the individual to critically assess their own results and, if necessary, seek expert advice.

Publication of your CIF in IUCr journals

A basic structural check has been run on your CIF. These basic checks will be run on all CIFs submitted for publication in IUCr journals (*Acta Crystallographica*, *Journal of Applied Crystallography*, *Journal of Synchrotron Radiation*); however, if you intend to submit to *Acta Crystallographica Section C* or *E* or *IUCrData*, you should make sure that full publication checks are run on the final version of your CIF prior to submission.

Publication of your CIF in other journals

Please refer to the *Notes for Authors* of the relevant journal for any special instructions relating to CIF submission.

PLATON version of 18/09/2020; check.def file version of 20/08/2020

Datablock liuhp_1106_3 - ellipsoid plot

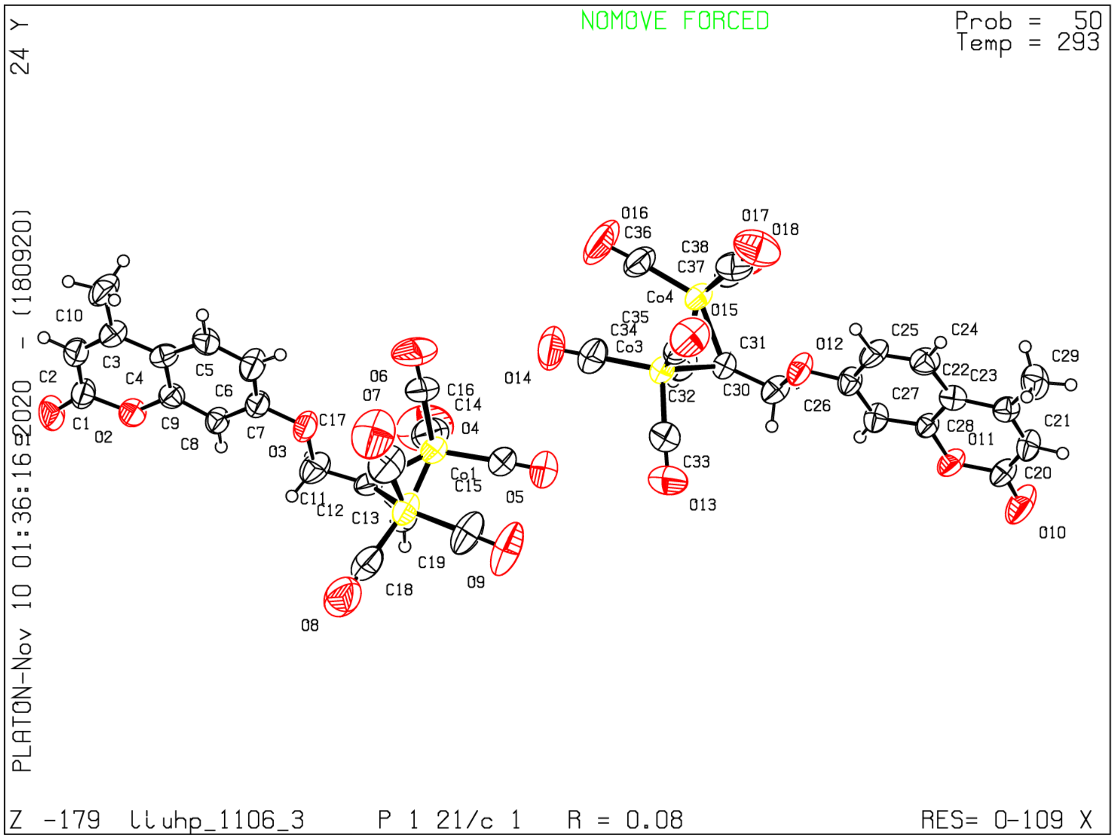

Supplement: Supplementary file 1 [file DataSheet2.ZIP › comp.5_cifreport.pdf]
