# Supplementary material for: Synthesis, docking studies, biological activity of carbon monoxide release molecules based on coumarin derivatives
Source: Front Chem. 2022 Sep 29;10:996079. doi: 10.3389/fchem.2022.996079 (PMC9557063; doi:10.3389/fchem.2022.996079)
Supplement: Supplementary file 2 [file DataSheet1.DOCX]

Supplementary Material

SI-Table 1 The details of the data collection and structure refinement.

| Complex | **5** |
| --- | --- |
| Formula | C_19_H_10_Co_2_O_9_ |
| Formula weight | 500.13 |
| Temperature/K | 293.19(12) |
| Crystal system | monoclinic |
| Dimensions(mm^3^) | 0.07×0.02×0.01 |
| Space group | *P*2_1_/c |
| a(Å) | 19.8707(9) |
| b(Å) | 7.3081(3) |
| c(Å) | 29.0760(14) |
| Volume(Å^3^) | 4055.1(3) |
| Z | 8 |
| D_calcd_ (mg/cm^3^) | 1.638 |
| *μ*(mm^-1^) | 13.267 |
| *F*(000) | 2000.0 |
| θ-range for data collection (°) | 4.4 to 70.1 |
| Reflections collected | 14295 |
| Independent reflections | 7481[R_int_= 0.076, R_sigma_=0.1289] |
| Data/restraints/parameter | 7481/0/543 |
| Final R-indices [*I* ≥ 2σ(*I*)] | R_1_=0.0806, wR_2_= 0.1522 |
| Final R indices [all data] | R_1_=0.1433, wR_2_= 0.1871 |
| Largest diff.peak and hole (e Å^-3^) | 0.36 / -0.36 |

SI-Table 2 The partial bond length and bond angle of complex **5**

| Bond lengths(Å) |  |  |  |
| --- | --- | --- | --- |
| Co(1)-Co(2) | 2.473(2) | Co(3)-Co(4) | 2.4615(19) |
| Co(1)-C(12) | 1.943(8) | Co(3)-C(31) | 1.961(8) |
| Co(2)-C(12) | 1.918(8) | Co(4)-C(31) | 1.938(7) |
| Co(2)-C(13) | 1.934(9) | Co(4)-C(32) | 1.965(9) |
| O(1)-C(1) | 1.194(10) | O(10)-C(20) | 1.216(9) |
| O(2)-C(1) | 1.387(9) | O(11)-C(20) | 1.404(9) |
| O(3)-C(11) | 1.444(9) | O(12)-C(30) | 1.428(9) |
| C(3)-C(4) | 1.458(10) | C(22)-C(23) | 1.449(10) |
| C(12)-C(13) | 1.338(11) | C(31)-C(32) | 1.331(11) |
| Bond angles(°) |  |  |  |
| C(12)-Co(1)-Co(2) | 49.7(2) | C(31)-Co(3)-Co(4) | 50.4(2) |
| C(12)-Co(1)-C(13) | 40.2(3) | C(32)-Co(3)-Co(4) | 51.3(3) |
| C(13)-Co(1)-Co(2) | 50.2(3) | C(32)-Co(3)-C(31) | 39.8(3) |
| C(14)-Co(1)-Co(2) | 146.2(4) | C(33)-Co(3)-Co(4) | 144.8(3) |
| C(14)-Co(1)-C(12) | 101.2(5) | C(33)-Co(3)-C(31) | 95.0(4) |
| C(12)-Co(2)-Co(1) | 50.6(2) | C(31)-Co(4)-Co(3) | 51.3(2) |
| C(12)-Co(2)-C(13) | 40.6(3) | C(31)-Co(4)-C(32) | 39.9(3) |
| C(13)-Co(2)-Co(1) | 50.6(2) | C(32)-Co(4)-Co(3) | 50.8(3) |
| C(17)-Co(2)-Co(1) | 99.8(4) | C(36)-Co(4)-Co(3) | 96.3(3) |
| C(9)-O(2)-C(1) | 123.1(6) | C(28)-O(11)-C(20) | 120.1(6) |
| O(1)-C(1)-O(2) | 117.5(8) | O(10)-C(20)-O(11) | 114.2(8) |
| O(1)-C(1)-C(2) | 127.2(8) | O(10)-C(20)-C(21) | 128.4(8) |
| O(2)-C(1)-C(2) | 115.3(7) | O(11)-C(20)-C(21) | 117.5(7) |
| Co(2)-C(12)-Co(1) | 79.6(3) | Co(4)-C(31)-Co(3) | 78.3(3) |
| Co(2)-C(13)-Co(1) | 79.2(3) | Co(3)-C(32)-Co(4) | 77.9(3) |
| C(12)-C(13)-Co(2) | 69.1(5) | C(31)-C(32)-Co(4) | 69.0(5) |
| O(9)-C(19)-Co(2) | 178.3(11) | O(18)-C(38)-Co(4) | 176.8(10) |

**
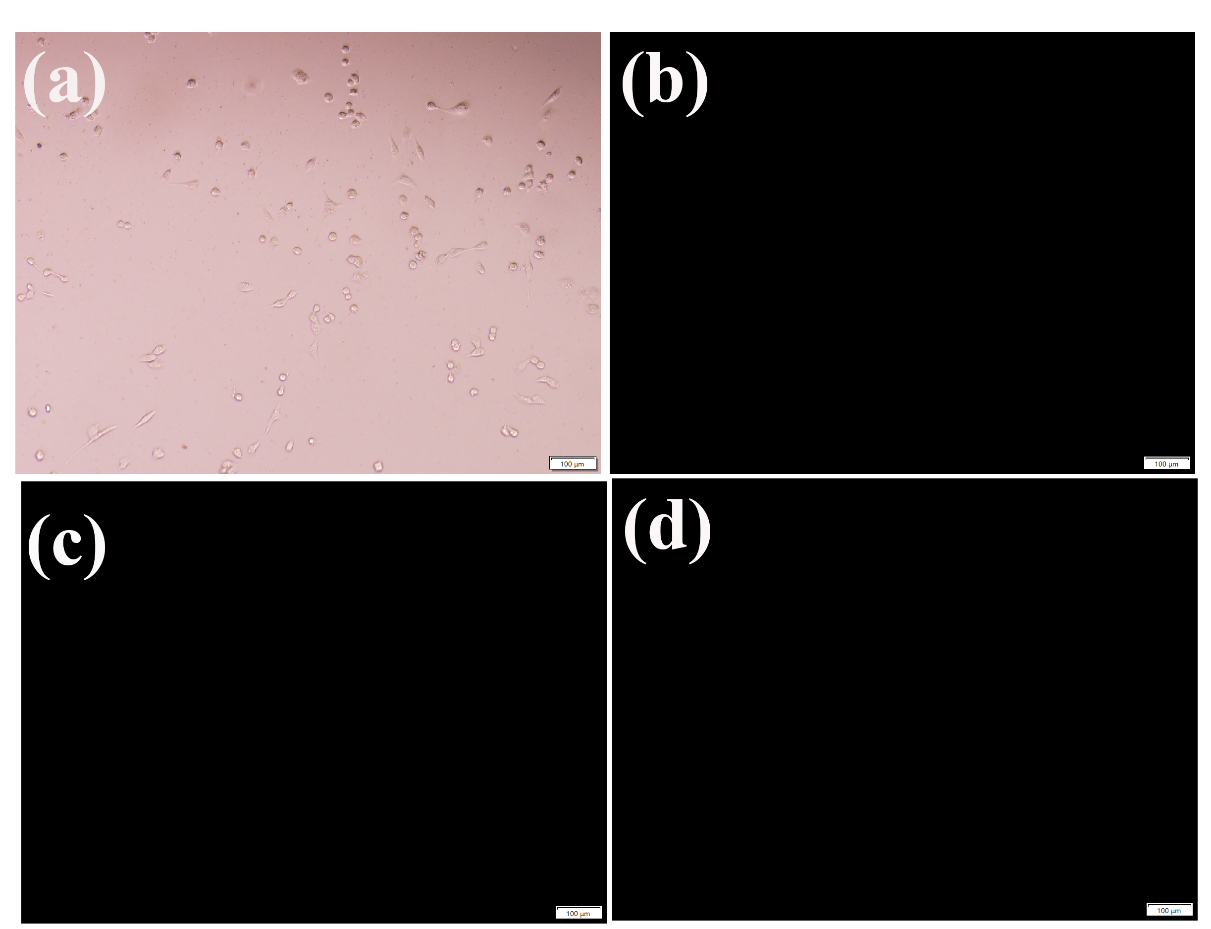
**

**SI-Figure 1.** The image of Tumor Cells MDAMB231 treated with **5** visualized by fluorescence microscope images of MDAMB231 cells. for 40 μM after treated with complex **5** for 24h**.**


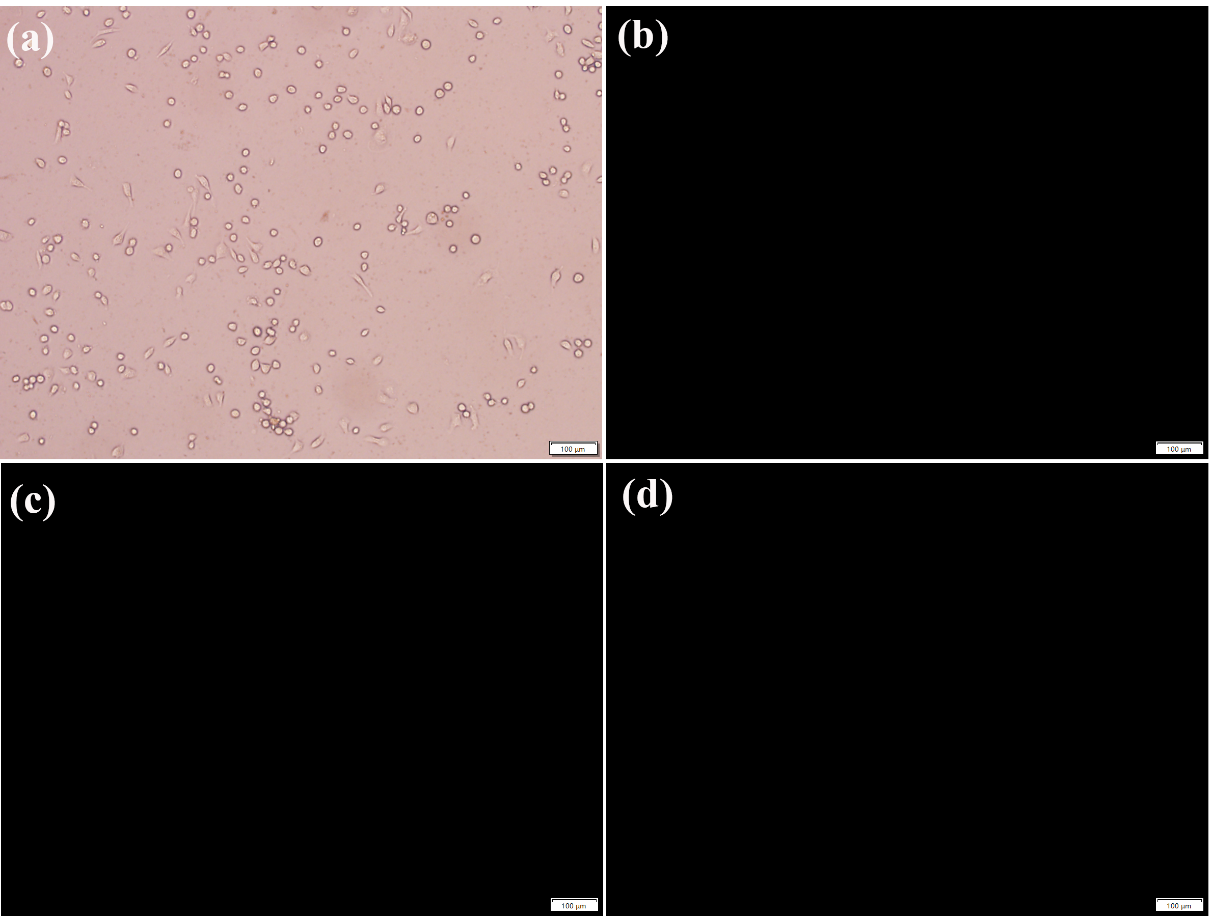


**SI-Figure 2** Image visualized with a fluorescence microscope. For 40 μM(d, e,f) after treated with complex **5** for 24h**.**

**^1^H****-NMR, ^19^F-NMR and ^13^C-NMR of the complexes**

The ^1^H-NMR spectra of **A1**

The ^13^C-NMR spectra of **A1**

The ^1^H-NMR spectra of **B1**

The ^13^C-NMR spectra of **B1**

The ^1^H-NMR spectra of **C1**

The ^13^C-NMR spectra of **C1**

The ^1^H-NMR spectra of **D1**

The ^13^C-NMR spectra of **D1**

The ^1^H-NMR spectra of **E1**

The ^13^C-NMR spectra of **E1**

The ^1^H-NMR spectra of **F1**

The ^13^C-NMR spectra of **F1**

The ^19^F-NMR spectra of **F1**

The ^1^H-NMR spectra of **G1**

The ^13^C-NMR spectra of **G1**

The ^19^F-NMR spectra of **G1**

The ^1^H-NMR spectra of complex **1**

The ^13^C-NMR spectra of complex **1**

The ^1^H-NMR spectra of complex **2**

The ^13^C-NMR spectra of complex **2**

The ^1^H-NMR spectra of complex **3**

The ^1^H-NMR spectra of complex **4**

The ^1^H-NMR spectra of complex **5**

The ^13^C-NMR spectra of complex **5**

The ^1^H-NMR spectra of complex **6**

The ^19^F-NMR spectra of complex **6**

The ^1^H-NMR spectra of complex **7**

The ^13^C-NMR spectra of complex **7**

The ^19^F-NMR spectra of complex **7**
